# Effect of high-flow nasal therapy on dyspnea, comfort, and respiratory rate

**DOI:** 10.1186/s13054-019-2473-y

**Published:** 2019-06-05

**Authors:** Andrea Cortegiani, Claudia Crimi, Alberto Noto, Yigal Helviz, Antonino Giarratano, Cesare Gregoretti, Sharon Einav

**Affiliations:** 1 Department of Surgical, Oncological and Oral Science (Di.Chir.On.S.), Section of Anesthesia, Analgesia, Intensive Care and Emergency, Policlinico Paolo Giaccone, University of Palermo, Via del vespro 129, 90127 Palermo, Italy; 2 Respiratory Medicine Unit, A.O.U. “Policlinico-Vittorio Emanuele”, Department of Clinical and Experimental Medicine, University of Catania, Via Santa Sofia 78, 95123 Catania, Italy; 3 Department of Anesthesia and Critical Care, A.O.U. Policlinico “G. Martino”, University of Messina, Via Consolare Valeria 1, 98100 Messina, Italy; 4 Intensive Care Unit of the Shaare Zedek Medical Medical Centre and Hebrew University Faculty of Medicine, Jerusalem, Israel

## Letter to the Editor

Systematic reviews comparing the effect of high-flow nasal treatment (HFNT) to conventional oxygen therapy (COT) or noninvasive ventilation (NIV) have focused on major clinical outcomes (i.e., endotracheal intubation, mortality) [1–3]. None have explored weaker outcomes that may nonetheless be important from the patient’s perspective, yet physiopathological mechanisms suggest that the HFNT may provide some advantage in this regard [4, 5]. We therefore systematically reviewed all randomized (RCTs) and crossover trials enrolling patients either post-extubation or during acute respiratory failure (ARF), comparing HFNT to COT or NIV and reporting data about dyspnea, comfort, and respiratory rate (RR) (PROSPERO CRD42019119536).

Full search strategy, detailed study methods, reference lists, and risk of bias assessments are reported in Additional file 1.

Twenty-four relevant studies were identified and included: for patients post-extubation, ten RCTs and one crossover trial and, for patients in ARF, eight RCTs and five crossover trials.

The summary of our findings is presented in the Table 1. More studies compared the effects of HNFT vs COT rather than vs NIV. Overall, there seems to be a trend showing that HFNT is probably not inferior to COT in most studies and perhaps better than NIV in terms of dyspnea, comfort, and decreasing of RR in some studies.

**Table 1:** Summary of findings in studies of the HFNT with regard to dyspnea, comfort, and respiratory rate

Study	Type	Design	Intervention (*N*)	Control (*N*)	Treatment methods	Measurement method	Dyspnea	Comfort	Respiratory rate
Bell N. [6] *Emerg Med Australas 2015*	AHRF	RCT	HFNT (48)	COT (52)	HFNT: flow 50 L/m, FiO2 30% titrated to SpO2 95% COT: discretion of the treating physician	Dyspnea: Borg Scale Comfort: Likert Scale	HFNT^§^	HFNT^§^ (1 h)	HFNT^§^ (2 h)
Frat J.P. [7] *N Engl J Med 2015*	AHRF	RCT	HFNT (106)	COT (94) NIV (110)	HFNT: flow 50 L/m, FiO2 100% then titrated to SpO2 92% COT: O2 titrated to SpO2 92% NIV: PSV PEEP from 2 up to 10 fiO2 adjusted to SpO2 92%	Dyspnea: Likert Scale Comfort: VAS	HFNT^§^	HFNT^§^	HFNT^§^ (1 h)
Lemiale V. [8] *Crit Care 2015*	AHRF (Immunocompromised)	RCT	HFNT (52)	COT (52)	HFNT: flow from 40 up to 50 L/m, FiO2 titrated to SpO2 95% COT: O2 titrated to SpO2 95%	Dyspnea: VAS Comfort: VAS	NS	NS	NS
Jones P.G. [9] *Respir Care 2016*	AHRF	RCT	HFNT (172)	COT (150)	HFNT: flow 40 L/m, 37 °C, FiO2 28% COT: FiO2 titrated to clinical needs	Dyspnea: Survey questions Comfort: Survey questions	NS	Overall comfort: NS “Dry my nose”: HFNT^§^ “In future I prefer”: COT^§^ “This method is worst”: HFNT^§^	NS
Doshi P. [10] *Ann Emergency Med 2017*	AHRF	RCT	HFNT (104)	NIV (112)	HFNT: flow from 35 L/m up to 40 L/m, T° between 35 and 37 °C NIV: IPAP from 10 up to 20 cmH20, EPAP from 5 up to 10 cmH20, FiO2 100%	Dyspnea: Borg Scale Comfort: NA	NA	NA	NA
Makdee O. [11] *Ann Emergency Med 2017*	AHRF (CPE)	RCT	HFNT (63)	COT (65)	HFNT: flow from 35 up to 60 L/m, FiO2 titrated to SpO2 95% COT: O2 titrated to SpO2 95%	Dyspnea: VAS Comfort: NA	NS	NA	HFNT^§^ (15, 30, 60 min)
Azoulay E. [12] *JAMA 2018*	AHRF (Immunocompromised)	RCT	HFNT (388)	COT (388)	HFNT: flow 50 L/min, FiO2 titrated to SpO2 95% COT: O2 titrated to SpO2 95%	Dyspnea: Dyspnea Score Comfort: VAS	NS	NS	HFNC^§^ (6 h)
Spoletini G. [13] *J Crit Care 2018*	AHRF (On NIV)	RCT	HFNT (23)	COT (24)	HFNT: flow 35 L/m, FiO2 titrated to SpO2 92% (hypoxic) or to 88–92%(hypercapnic) COT: flow adjusted to maintain the same SpO2	Dyspnea: Borg Scale Comfort: VAS	NS	HFNT^§^	NS
Cuquemelle E. [14] *Respir Care 2012*	AHRF	Crossover	HFNT (37)	COT (37)	HFNT: flow 40 L/m, FiO2 titrated to SpO2 95% COT: O2 titrated to SpO2 95%	Dyspnea: NA Comfort: Dryness	NA	HFNT^§^	NA
Schwabbauer N. [15] *BMC Anesthesiol 2014*	AHRF	Crossover	HFNT (14)	COT (14) NIV (14)	HFNT: flow 55 L/m, FiO2 60% COT: Venturi mask FiO2 60% NIV: PSV FiO2 60% PEEP 5 cmH20 PS 6-8 ml/kg PBW	Dyspnea: Borg Scale Comfort: NRS	HFNT vs. COT HFNT^§^ vs. NIV	HFNT vs. COT HFNT^§^ vs. NIV	HFNT vs. COT HFNT vs. NIV COT vs. NIV^§^
Vargas F. [16] *Respir Care 2015*	AHRF	Crossover	HFNT (*n* = 12)	COT (12) CPAP (12)	HFNT: flow 60 L/m, T 37 °C, FiO2 same as COT COT: O2 titrated to SpO2 90% CPAP: 5 cmH20 FiO2 same as COT	Dyspnea: Dyspnea Score Comfort: NRS	NS	NS	HFNT^§^ vs. COT HFNT vs. CPAP
Mauri T. [17] *Am J Respir Crit Care Med 2017*	AHRF	Crossover	HFNT (15)	COT (15)	HFNT: flow 40 L/m, FiO2 titrated to SpO2 90–95% COT: Airvo2 face mask 12 L/min same FiO2	Dyspnea: DeltaPes Comfort: NA	HFNT^§^	NA	HFNT^§^
Sklar M.C. [18] *Ann Intensive Care 2018*	ARF (Exacerbation of cystic fibrosis)	Crossover	HFNT (15)	NIV (15)	HFNT: flow 55 L/m, T° 34 or 37 °C FiO2 titrated to SpO2 92% NIV: FiO2 titrated to SpO2 92%, setting as previously adjusted	Dyspnea: VAS Comfort: VAS	NS	NS	NS
Parke R. [19] *Br J Anaesth 2013*	Post-extubation (Cardiac surgery)	RCT	HFNT (169)	COT (171)	HFNT: flow 45 L/m, FiO2 titrated to SpO2 93% COT: O2 titrated to SpO2 93%	Dyspnea: NA Comfort: NRS	NA	HFNT^§^	NA
Maggiore S.M. [20] *Am J Respir Crit Care Med 2014*	Post-extubation	RCT	HFNT (53)	COT (52)	HFNT: flow 50 L/m, FiO2 titrated to SpO2 92–98% (hypoxic) or to 88–95%(hypercapnic) COT: O2 titrated to SpO2 92–98% (hypoxic) or 88–95%(hypercapnic)	Dyspnea: NA Comfort: NRS	NA	Interface: HFNT^§^ (from 12 h) Dryness: HFNT^§^ (from 24 h)	HFNT^§^ (from 1 h)
Corley A. [21] *Intensive Care Med 2015*	Post-extubation (Cardiac)	RCT	HFNT (81)	COT (74)	HFNT: flow 35 up to 50 L/min, T 37 °C, FiO2 titrated to SpO2 95% COT: O2 titrated to SpO2 95%	Dyspnea: Borg Scale Comfort: NA	COT^§^ (8 h)	NA	NS
Stephan F. [22] *JAMA 2015*	Post-extubation (Cardiac)	RCT	HFNT (414)	NIV (416)	HFNT: flow 50 L/m, FiO2 titrated to SpO2 92–98% NIV: PEEP and PS adjusted to RR < 25/min and TV 8 ml/kg, FiO2 SpO2 92–98%	Dyspnea: Dyspnea Score Comfort: NRS	NS	NS	HFNT^§^(1 h, 1 day, 2 days, 3 days)
Futier E. [23] *Intensive Care Med 2016*	Post-extubation (Abdominal or thoracic)	RCT	HFNT (108)	COT (112)	HFNT: flow 50–60 L/m, FiO2 titrated to SpO2 95% COT: O2 titrated to SpO2 95%	Dyspnea: NA Comfort: NRS	NA	NS	NA
Hernandez G. (a) [24] *JAMA 2016*	Post-extubation (Low-risk extubation failure)	RCT	HFNT (264)	COT (263)	HFNT: flow 10 L/m titrated in 5 L step until discomfort, FiO2 to SpO2 92%, T 37 °C COT: O2 titrated to SpO2 92%	Dyspnea: NA Comfort: NA	NA	NA	NA
Hernandez G. (b) [25] *JAMA 2016*	Post-extubation (High-risk extubation failure)	RCT	HFNT (290)	NIV (314)	HFNT: flow 10 L/m titrated in 5 L step until discomfort, FiO2 to SpO2 92%, T 37 °C NIV: PEEP and PS adjusted to RR 25/min, SpO2 92%, pH 7.35	Dyspnea: NA Comfort: NA	NA	NA	NA
Fernandez R. [26] *Ann Intensive Care 2017*	Post-extubation (High-risk extubation failure)	RCT	HFNT (78)	COT (77)	HFNT: flow 40 L/min (adjusted on tolerance), T 37 or 34 °C, FiO2 titrated to SpO2 92–95% COT: O2 titrated to SpO2 92–95%	Dyspnea: NA Comfort: NA	NA	NA	NA
Yu Y. [27] *Can Respir J 2017*	Post-extubation (Thoracic)	RCT	HFNT (56)	COT (54)	HFNT: flow from 35 to 60 L/m, FiO2 titrated to SpO2 95% COT: O2 titrated to SpO2 95%	Dyspnea: NA Comfort: Rates of throat/nasal pain	NA	HFNT^§^	HFNT^§^ (1 h, 2 h, 6 h, 24 h, 48 h, 72 h)
Song H.Z. [28] *Clinics (Sao Paulo) 2017*	post-extubation	RCT	HFNT (30)	COT (30)	HFNT: flow 60 L/m, FiO2 titrated to SpO2 94–98% (hypoxic) or to 88–92% (hypercapnic) COT: O2 titrated to SpO2 94–98% (hypoxic) or 88–92%(hypercapnic)	Dyspnea: NA Comfort: VAS	NA	HFNT^§^ (interface) HFNT^§^ (dryness)	HFNT^§^
Rittayamai N. [29] *Respir Care 2014*	post-extubation	Crossover	HFNT (17)	COT (17)	HFNT: flow 35 L/m, FiO2 titrated to SpO2 94% COT: O2 titrated to SpO2 94%	Dyspnea: VAS Comfort: VAS	HFNT^§^ (10, 15, 30 min)	NS	HFNT^§^ (5, 10, 15, 30 min)

*AHFR* acute hypoxemic respiratory failure, *ARF* acute respiratory failure, *CPAP* continuous positive airway pressure, *COT* conventional oxygen therapy, *HFNT* high-flow nasal treatment, *h* hours, *IPAP* inspiratory positive airway pressure, *N* number of patients, *NA* not available, *NIV* noninvasive ventilation, *NS* not statistically significant, *PES* esophageal pressure, *PSV* pressure support ventilation, *RCT* randomized controlled trial, *VAS* visual analog scale

^§^Comparison between intervention and control with a statistically significant *p* value in favor of (the specified intervention)

Heterogeneity in case-mix, the tools used for outcome assessment and measurement time-points precluded performance of meta-analysis. Neither patients nor treating clinicians were blinded to the intervention in any of the trials, introducing a high risk of detection bias. Differences in HFNT settings (i.e., flow and temperature) and a lack of full description for weaning criteria or protocol may have also contributed to the diversity in findings with regard to comfort and dyspnea.

In this analysis of the literature, the use of HFNT during ARF or post-extubation seems to be not clearly associated with improvements in comfort, dyspnea, and RR since findings from the most recent available evidence were inconsistent. However, in this regard, HFNT does not seem inferior to either COT or NIV. Future research should be focused in assessing patient-reported outcomes using appropriate standardized and validated measures in order to investigate the comparative effectiveness of the different respiratory support strategies.

## Additional file

Additional file 1: List of included studies, search strategy, and risk of bias assessment. Detailed study methods, reference list of included studies, search strategy, risk of bias assessment. (DOCX 520 kb)

## Abbreviations

ARF: Acute respiratory failure
COT: Conventional oxygen therapy
HFNT: High-flow nasal therapy
NIV: Noninvasive ventilation
RCT: Randomized controlled trial
RR: Respiratory rate
