# Supplementary material for: Effect of high-flow nasal therapy on dyspnea, comfort, and respiratory rate
Source: Crit Care. 2019 Jun 5;23:201. doi: 10.1186/s13054-019-2473-y (PMC6549315; doi:10.1186/s13054-019-2473-y)
Supplement: Supplementary file 1 — List of included studies, search strategy, and risk of bias assessment. Detailed study methods, reference list of included studies, search strategy, risk of bias assessment. (DOCX 520 kb) [file 13054_2019_2473_MOESM1_ESM.docx]

**Effect of High Flow Nasal Therapy on Dyspnea, Comfort and Respiratory Rate: a Systematic Review**

METHODS

**Data Sources and Searches**

The study was registered in PROSPERO (CRD42019119536). We performed a comprehensive search of MEDLINE and EMBASE. The last date of the search was 06 January 2019. We used the key words “*humans*” together with “*adult*”, “*mature*” or “*grown*”. Publications with the key words “*high flow nasal cannula*”, “*high flow nasal therapy*”, “*high flow nasal oxygen*”, “*high flow oxygen therapy*”, “*high flow therapy*”, “*optiflow (respiration)*” and “*nasal highflow*”. We restricted the search to studies published in the English and Spanish language. We did not perform an online search for additional conference proceedings or abstracts.

**Study selection**

The content experts performed screening in two stages, initially assessing titles and abstracts, and then the full articles for those possibly eligible. Both stages of screening were performed in duplicate. Two of the authors (AC and YH) screened for papers relevant to the topics of this systematic review (dyspnea, comfort, respiratory rate). Disagreements were resolved by discussion with two third parties (CG and SE) with common adjudication when required. AC and YH also screened all potentially relevant citations and references. Where relevant, the reasons for exclusion were captured at the stage of full article review.

We included all cross-over, and randomized studies comparing the use of HFNT (study group) to COT, NIV or CPAP (control groups). We excluded case reports, case series and observational studies. The population of interest were critically ill adult patients either post-extubation or with acute hypoxemic respiratory failure (defined as such by the authors). We included the following outcomes: dyspnea (any definition), comfort (any definition), respiratory rate (breaths per minute) where available.

**Data Extraction and Quality Assessment**

Two of the reviewers (CC and AN) performed data extraction independently and in duplicate using predefined data abstraction tables. Third parties (AC and SE) resolved disagreements. Abstracted data included study reference, details of the intervention and control, primary and secondary outcome data, and risk of bias (RoB) for each study RoB was assessed by an author with appropriate training (AC) by outcome per study using a Cochrane RoB tool that classifies RoB as “low,” “unclear” and “high” for each of the following domains: sequence generation, allocation sequence concealment, blinding, selective outcome reporting, and other bias. We rated the overall RoB as the highest risk attributed to any criterion. We did not assess the overall certainty of evidence for each outcome using the Grading of Recommendations Assessment, Development and Evaluation (GRADE) framework due to the high RoB found. Due to heterogeneity between studies in populations included and comparators, the scales used to measure the outcomes and lack of details regarding the intervention, a decision was made not perform meta-analysis of the data.

**LIST OF INCLUDED STUDIES**

1. Azoulay E, Lemiale V, Mokart D, Nseir S, Argaud L, Pene F, Kontar L, Bruneel F, Klouche K, Barbier F, Reignier J, Berrahil-Meksen L, Louis G, Constantin JM, Mayaux J, Wallet F, Kouatchet A, Peigne V, Theodose I, Perez P, Girault C, Jaber S, Oziel J, Nyunga M, Terzi N, Bouadma L, Lebert C, Lautrette A, Bige N, Raphalen JH, Papazian L, Darmon M, Chevret S, Demoule A, (2018) Effect of High-Flow Nasal Oxygen vs Standard Oxygen on 28-Day Mortality in Immunocompromised Patients With Acute Respiratory Failure: The HIGH Randomized Clinical Trial. JAMA 320: 2099-2107

2. Bell N, Hutchinson CL, Green TC, Rogan E, Bein KJ, Dinh MM, (2015) Randomised control trial of humidified high flow nasal cannulae versus standard oxygen in the emergency department. Emerg Med Australas 27: 537-541

3. Corley A, Bull T, Spooner AJ, Barnett AG, Fraser JF, (2015) Direct extubation onto high-flow nasal cannulae post-cardiac surgery versus standard treatment in patients with a BMI >/=30: a randomised controlled trial. Intensive Care Med 41: 887-894

4. Cuquemelle E, Pham T, Papon JF, Louis B, Danin PE, Brochard L, (2012) Heated and humidified high-flow oxygen therapy reduces discomfort during hypoxemic respiratory failure. Respir Care 57: 1571-1577

5. Doshi P, Whittle JS, Bublewicz M, Kearney J, Ashe T, Graham R, Salazar S, Ellis TW, Jr., Maynard D, Dennis R, Tillotson A, Hill M, Granado M, Gordon N, Dunlap C, Spivey S, Miller TL, (2018) High-Velocity Nasal Insufflation in the Treatment of Respiratory Failure: A Randomized Clinical Trial. Ann Emerg Med 72: 73-83 e75

6. Fernandez R, Subira C, Frutos-Vivar F, Rialp G, Laborda C, Masclans JR, Lesmes A, Panadero L, Hernandez G, (2017) High-flow nasal cannula to prevent postextubation respiratory failure in high-risk non-hypercapnic patients: a randomized multicenter trial. Ann Intensive Care 7: 47

7. Frat JP, Thille AW, Mercat A, Girault C, Ragot S, Perbet S, Prat G, Boulain T, Morawiec E, Cottereau A, Devaquet J, Nseir S, Razazi K, Mira JP, Argaud L, Chakarian JC, Ricard JD, Wittebole X, Chevalier S, Herbland A, Fartoukh M, Constantin JM, Tonnelier JM, Pierrot M, Mathonnet A, Beduneau G, Deletage-Metreau C, Richard JC, Brochard L, Robert R, Group FS, Network R, (2015) High-flow oxygen through nasal cannula in acute hypoxemic respiratory failure. N Engl J Med 372: 2185-2196

8. Futier E, Paugam-Burtz C, Godet T, Khoy-Ear L, Rozencwajg S, Delay JM, Verzilli D, Dupuis J, Chanques G, Bazin JE, Constantin JM, Pereira B, Jaber S, investigators Os, (2016) Effect of early postextubation high-flow nasal cannula vs conventional oxygen therapy on hypoxaemia in patients after major abdominal surgery: a French multicentre randomised controlled trial (OPERA). Intensive Care Med 42: 1888-1898

9. Hernandez G, Vaquero C, Colinas L, Cuena R, Gonzalez P, Canabal A, Sanchez S, Rodriguez ML, Villasclaras A, Fernandez R, (2016) Effect of Postextubation High-Flow Nasal Cannula vs Noninvasive Ventilation on Reintubation and Postextubation Respiratory Failure in High-Risk Patients: A Randomized Clinical Trial. JAMA 316: 1565-1574

10. Hernandez G, Vaquero C, Gonzalez P, Subira C, Frutos-Vivar F, Rialp G, Laborda C, Colinas L, Cuena R, Fernandez R, (2016) Effect of Postextubation High-Flow Nasal Cannula vs Conventional Oxygen Therapy on Reintubation in Low-Risk Patients: A Randomized Clinical Trial. JAMA 315: 1354-1361

11. Jones PG, Kamona S, Doran O, Sawtell F, Wilsher M, (2016) Randomized Controlled Trial of Humidified High-Flow Nasal Oxygen for Acute Respiratory Distress in the Emergency Department: The HOT-ER Study. Respir Care 61: 291-299

12. Lemiale V, Mokart D, Mayaux J, Lambert J, Rabbat A, Demoule A, Azoulay E, (2015) The effects of a 2-h trial of high-flow oxygen by nasal cannula versus Venturi mask in immunocompromised patients with hypoxemic acute respiratory failure: a multicenter randomized trial. Crit Care 19: 380

13. Maggiore SM, Idone FA, Vaschetto R, Festa R, Cataldo A, Antonicelli F, Montini L, De Gaetano A, Navalesi P, Antonelli M, (2014) Nasal high-flow versus Venturi mask oxygen therapy after extubation. Effects on oxygenation, comfort, and clinical outcome. Am J Respir Crit Care Med 190: 282-288

14. Makdee O, Monsomboon A, Surabenjawong U, Praphruetkit N, Chaisirin W, Chakorn T, Permpikul C, Thiravit P, Nakornchai T, (2017) High-Flow Nasal Cannula Versus Conventional Oxygen Therapy in Emergency Department Patients With Cardiogenic Pulmonary Edema: A Randomized Controlled Trial. Ann Emerg Med 70: 465-472 e462

15. Mauri T, Turrini C, Eronia N, Grasselli G, Volta CA, Bellani G, Pesenti A, (2017) Physiologic Effects of High-Flow Nasal Cannula in Acute Hypoxemic Respiratory Failure. Am J Respir Crit Care Med 195: 1207-1215

16. Parke R, McGuinness S, Dixon R, Jull A, (2013) Open-label, phase II study of routine high-flow nasal oxygen therapy in cardiac surgical patients. Br J Anaesth 111: 925-931

17. Rittayamai N, Tscheikuna J, Rujiwit P, (2014) High-flow nasal cannula versus conventional oxygen therapy after endotracheal extubation: a randomized crossover physiologic study. Respir Care 59: 485-490

18. Schwabbauer N, Berg B, Blumenstock G, Haap M, Hetzel J, Riessen R, (2014) Nasal high-flow oxygen therapy in patients with hypoxic respiratory failure: effect on functional and subjective respiratory parameters compared to conventional oxygen therapy and non-invasive ventilation (NIV). BMC Anesthesiol 14: 66

19. Sklar MC, Dres M, Rittayamai N, West B, Grieco DL, Telias I, Junhasavasdikul D, Rauseo M, Pham T, Madotto F, Campbell C, Tullis E, Brochard L, (2018) High-flow nasal oxygen versus noninvasive ventilation in adult patients with cystic fibrosis: a randomized crossover physiological study. Ann Intensive Care 8: 85

20. Song HZ, Gu JX, Xiu HQ, Cui W, Zhang GS, (2017) The value of high-flow nasal cannula oxygen therapy after extubation in patients with acute respiratory failure. Clinics (Sao Paulo) 72: 562-567

21. Spoletini G, Mega C, Pisani L, Alotaibi M, Khoja A, Price LL, Blasi F, Nava S, Hill NS, (2018) High-flow nasal therapy vs standard oxygen during breaks off noninvasive ventilation for acute respiratory failure: A pilot randomized controlled trial. J Crit Care 48: 418-425

22. Stephan F, Barrucand B, Petit P, Rezaiguia-Delclaux S, Medard A, Delannoy B, Cosserant B, Flicoteaux G, Imbert A, Pilorge C, Berard L, Bi POPSG, (2015) High-Flow Nasal Oxygen vs Noninvasive Positive Airway Pressure in Hypoxemic Patients After Cardiothoracic Surgery: A Randomized Clinical Trial. JAMA 313: 2331-2339

23. Vargas F, Saint-Leger M, Boyer A, Bui NH, Hilbert G, (2015) Physiologic Effects of High-Flow Nasal Cannula Oxygen in Critical Care Subjects. Respir Care 60: 1369-1376

24. Yu Y, Qian X, Liu C, Zhu C, (2017) Effect of High-Flow Nasal Cannula versus Conventional Oxygen Therapy for Patients with Thoracoscopic Lobectomy after Extubation. Can Respir J 2017: 7894631

**Figure S1**

PRISMA flow chart of the systematic search. AHRF: acute respiratory failure; RCT: randomized controlled trial

**Figure S2**

Risk of bias assessment using the Cochrane risk of bias assessment tool. Green: low risk of bias; Yellow: unclear risk of bias; Red: high risk of bias
